# A new method for analyzing glistenings in hydrophobic acrylic intraocular lenses

**DOI:** 10.1038/s41598-025-00137-9

**Published:** 2025-05-21

**Authors:** M. Canberk Göktepe, Timur M. Yildirim, Grzegorz Łabuz, Thibaut Devogelaere, Gerd U. Auffarth, Ramin Khoramnia

**Affiliations:** 1 Department of Ophthalmology, The David J Apple Center for Vision Research, University of Heidelberg, Im Neuenheimer Feld 400, 69120 Heidelberg, Germany; 2 Devogelaere Vision, Oudenburg, Belgium; 3 Department of Ophthalmology, Faculty of Medicine and University Hospital Carl Gustav Carus, Technical University Dresden, Fetscherstraße 74, 01307 Dresden, Germany

**Keywords:** Intraocular lenses, Glistenings, Light microscopy, Optical coherence tomography, Straylight, Optical techniques, Translational research

## Abstract

A reproducible, examiner-independent, objective method was introduced that uses swept-source optical coherence tomography (SS-OCT) and compared with established methods. Glistenings were induced in-vitro in twenty-five IOLs, five different IOL models: AcrySof SN60WF, AcrySof MA60AC, Clareon CNA0T0, PY60AD and Vivinex XY1. We calculated the glistening density within each IOL using light microscopy and SS-OCT. Glare effects were quantified with a straylight meter device (Oculus C-Quant). Image-processing software provided an objective tool for glistening quantification. Correlation parameters were evaluated between results of the three methods by using linear regression analysis. The PY60AD showed the highest amount of glistenings, followed by the MA60AC and SN60WF, whereas the CNA0T0 and XY1 showed the lowest values. There was a moderate correlation (R^2^ = 0.80) between the light microscopy and the straylight method. The analysis also showed a moderate correlation (R^2^ = 0.77) between the SS-OCT and the straylight method. At the same time, we found a strong and proportional relationship (R^2^ = 0.91) between the in-vitro reference method light microscopy and the SS-OCT method. We propose that SS-OCT can analyze glistenings precisely and allow objective estimation of the straylight induced by the glistenings within an IOL, permitting the clinician to measure pseudophakic patients’ glare symptoms.

**Supplementary Information:**

The online version contains supplementary material available at 10.1038/s41598-025-00137-9.

## Introduction

The appearance of fluid-filled microvacuoles, so-called glistenings, in intraocular lenses (IOLs) is a common postoperative finding and mostly associated with hydrophobic acrylic IOLs^1,2^. Although glistening formation is less common in recent IOL materials, due to improvements in the manufacturing process, they still remain a relevant phenomenon in some materials^3,4^. Recent studies have shown that a significant amount of glistenings can induce forward light scattering, causing glare symptoms^5–8^.

Different methods such as slit lamp microscopy, Scheimpflug tomography, straylight measurements or light microscopy have been utilized in search for an examiner-independent, objective and reproducible method to quantify glistenings. However, studies show that all these methods are proven to have limitations in that regard^9–16^. An approach has just recently been suggested by Fernandez-Vigo et al. to quantify glistenings using swept-source optical coherence tomography (SS-OCT). In this clinical study, SS-OCT scans of pseudophakic patients were evaluated by two observers and a glistening classification system was proposed^17^. Although this study claims an approach for objective classification of glistenings in IOL, it has a limitation, since the proposed evaluation method still requires an observer. Recently, the same group proposed an automated approach using OCT and a deep learning (DL) based quantification algorithm. The new approach was compared with manual quantification of an expert. The study found a high agreement between the DL and expert assessment. However, the OCT based method was not compared with other established methods. The authors concluded that further studies are needed to evaluate the clinical correspondence between the OCT-based classification and functional tests^18^.

So far, there has yet to be a study that compares SS-OCT-based classification methods with established methods such as light microscopy and straylight measurements. The purpose of this study was to introduce a novel, objective, examiner-independent, reproducible method to quantify glistenings using a high-resolution SS-OCT device and compare it with established methods.

## Methods

### Accelerated aging procedure

In this experimental study, 25 hydrophobic acrylic IOLs from 5 different IOL models such as AcrySof SN60WF (Expiry Date: 2025-09-30), AcrySof MA60 AC (Expiry Date: 2025-12-15), Clareon CNA0T0 (Expiry Date: 2024-01-20) (Alcon Laboratory, Forth Worth, USA), PY60AD (Expiry Date: 2024-02-29) (Hoya, Tokyo, Japan) and Vivinex XY1 (Expiry Date: 2023-11-30) (Hoya, Tokyo, Japan) were used. In 25 IOLs (five of each model and all labelled with + 20.0 D power), glistenings were induced in-vitro using an accelerated aging method described previously^3^. Briefly, the IOLs were subjected to elevated temperature at 45 °C in a heat chamber for 24 h within vials filled with a sodium chloride 0,9% solution, then transferred to a water bath with a temperature of 37 °C for 2.5 h^3^ All methods of the study were performed in conformance with the tenets of the Declaration of Helsinki and German federal and state laws. This study solely involves laboratory analysis of non-organic materials (intraocular lenses). No procedures on animals/humans or animal/human tissue samples were performed. An approval of the experimental protocols was therefore not required.

### Glistening assessment

**Fig. 1:**
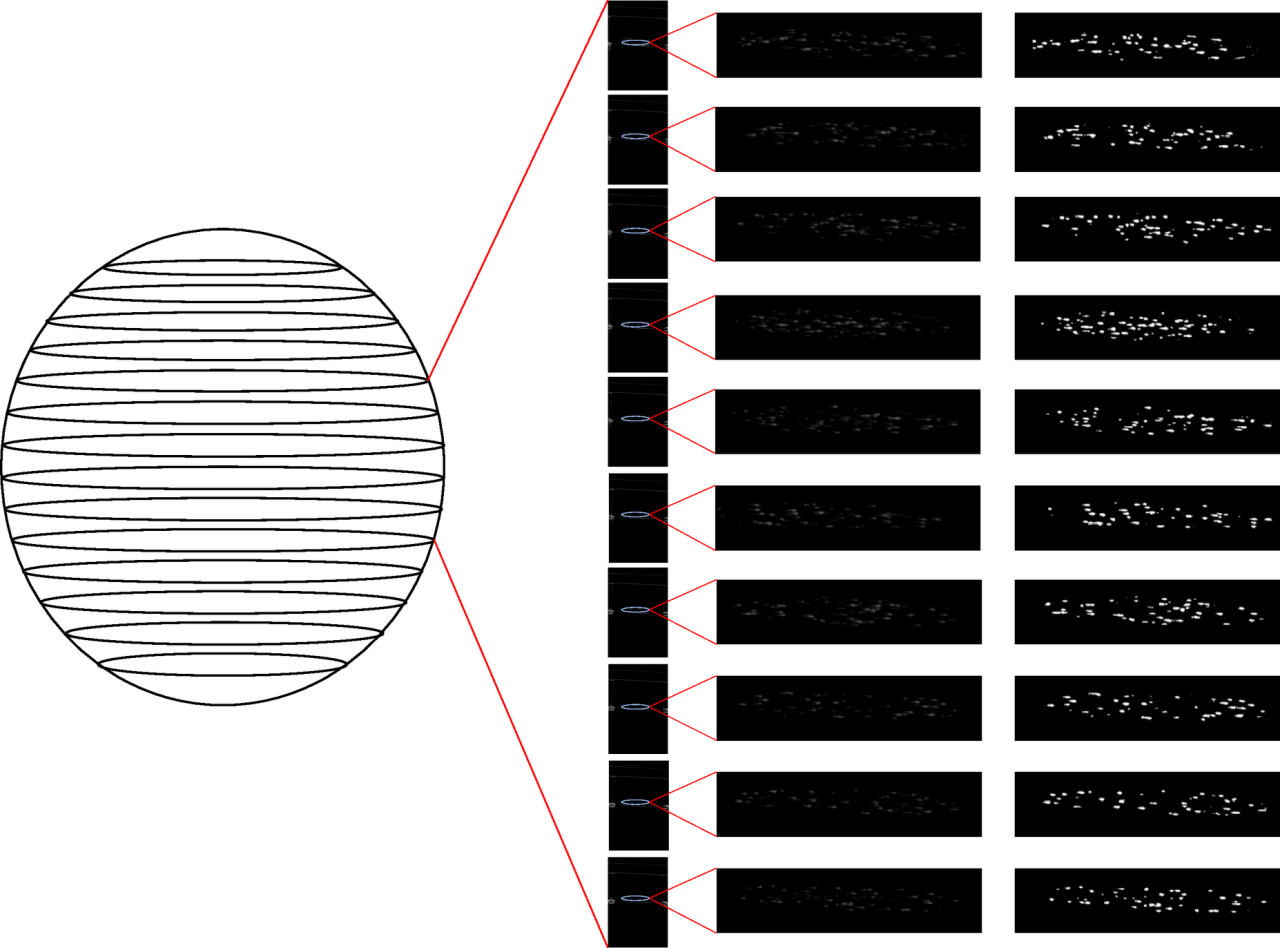
Image processing steps used to evaluate swept-source optical coherence tomography images. Sixty-five B-Scans of each IOL were obtained using the Imaging App and with the volume scan mode of an Anterion (Heidelberg Engineering. Heidelberg. Germany). B-Scan Angle was 0^0^. Volume height and volume width were 6 mm each. Scan resolution level was 1024. In this figure less than 65 B-Scans are shown for simplification purposes. Ten B-Scans of the central part of the IOL were chosen to quantify the amount of glistenings. A 1 mm^2^ area in the middle of each B-Scan was chosen as the region of interest (ROI). The ROI was segmented with Trainable Weka Segmentation and dichotomized with a threshold value. Following binarization. Glistenings were counted automatically by the software (ImageJ including Fiji toolkit version 1.53 t, National Institutes of Health, Bethesda, USA). Then, the mean glistening density was calculated for each IOL.

Observations were made on each IOL using different methods: light and dark-field microscopy (Meiji Techno, Saitama, Japan), SS-OCT using the Anterion (Heidelberg Engineering GmbH, Heidelberg, Germany) and straylight measurement using the C-Quant (Oculus Optikgeräte GmbH, Wetzlar, Germany). Dark-field microscopy was only used to obtain an overview image of an IOL, not for the quantification of glistenings. In between the experiments, the IOLs were put in an incubator, which was preheated to 37 °C to minimize the temperature changes to the IOL, thus preventing fluctuation in glistening density. A BX50 light microscope (Olympus, Tokyo, Japan) was used for morphological analysis, taking photographs from the central part of each IOL with 100x magnification. The images were calibrated with a microscope-stage micrometer. Image processing was performed using ImageJ including Fiji toolkit version 1.53 t (National Institutes of Health, Bethesda, USA) with the plugin Trainable Weka Segmentation (TWS). TWS is a pixel classifier, which combines the image processing toolkit Fiji with machine-learning algorithms provided in data mining and machine learning toolkit Waikato Environment for Knowledge Analysis (WEKA)^19^. For evaluation of images with this method, a classifier was trained by using a light microscopy image of an AcrySof IOL with glistenings. Two classes named “glistenings” and “background” were created. Glistenings and background were selected manually and labelled under the appropriate class. Once the training is completed, the trained classifier was used to segment the light microscopy image of each IOL. Subsequently, the segmented image was binarized using a predefined threshold value and the number of glistenings was counted automatically by the software. After the count, glistening density (Microvacuoles (MV)/mm^2^) was calculated. Additionally, the image processing program determined the average glistening size for each IOL. After the images of the IOL were obtained with light microscopy, the lens was transferred to a custom-made IOL holder for the second morphological examination method, which was the SS-OCT device. The IOL holder was built in cooperation with the Department of Precision Engineering of the Heidelberg University to examine IOLs in-vitro with the SS-OCT device^20^. It consists of a glass window and an artificial anterior chamber filled with a preheated (37 °C) sodium chloride 0,9% solution, in which the IOL was placed. The IOLs were examined with a Volume Scan Pattern. Sixty-five horizontal B-Scans along the IOL were obtained with a volume height and a B-Scan length of 6 mm. For the quantification of glistenings, 10 B-Scans in the central part of the lens were processed once again using ImageJ including Fiji toolkit version 1.53 t with the plugin TWS. A TWS classifier was trained beforehand using SS-OCT B-Scans of an AcrySof IOL with in-vitro induced glistenings. The classifier was trained in a similar manner to the one described in the light microscopy method. Two classes were set with the names “glistenings” and “background”. Glistenings and background were selected and labelled under the appropriate class. SS-OCT images were calibrated using the width of a central B-Scan, since the selected volume width was 6 mm. A 1 mm^2^ ellipsoid area was chosen as a region of interest (ROI) in the middle of each B-Scan. ROI was then segmented with the trained TWS classifier. After the segmentation, a fixed threshold was used to binarize each image. The number of glistenings was counted automatically and the glistening density (MV/mm^2^) was calculated. Figure 1shows these image processing steps in detail. Once the morphological analysis of each IOL with the SS-OCT device was completed, straylight measurements were carried out with the C-Quant straylight meter. The C-Quant device expresses the straylight parameter “s” as log(s) in a logarithmic scale. It was adapted to evaluate the straylight of IOLs in-vitro, as described in a previously published study^21^: after adaptation of the device, the IOL was placed in a custom-made IOL holder, which was then inserted in a wet-cell. The wet-cell was filled with preheated (37 °C) sodium chloride 0.9% solution and positioned in a rectangular component. With this setup, straylight measurements were conducted with and without the IOL. The straylight measurements without the IOL was performed to determine the straylight of the setup itself. Three consecutive straylight measurements were executed for each condition. The mean value of the three measurements was calculated and used in the following formula for the calculation of straylight induced by the IOL (S_IOL_):

$${\text{S}}_{{{\text{IOL}}}} \left( {{\text{deg}}^{{\text{2}}} /{\text{sr}}} \right){\text{ }} = {\text{ 1}}0^{{{\text{log}}({\text{S}}({\text{setup}} + {\text{IOL}}))}} {-}{\text{ 1}}0^{{{\text{log}}\left( {{\text{S}}\left( {{\text{Setup}}} \right)} \right)}}$$

### Analysis of explanted IOLs

**Fig. 2:**
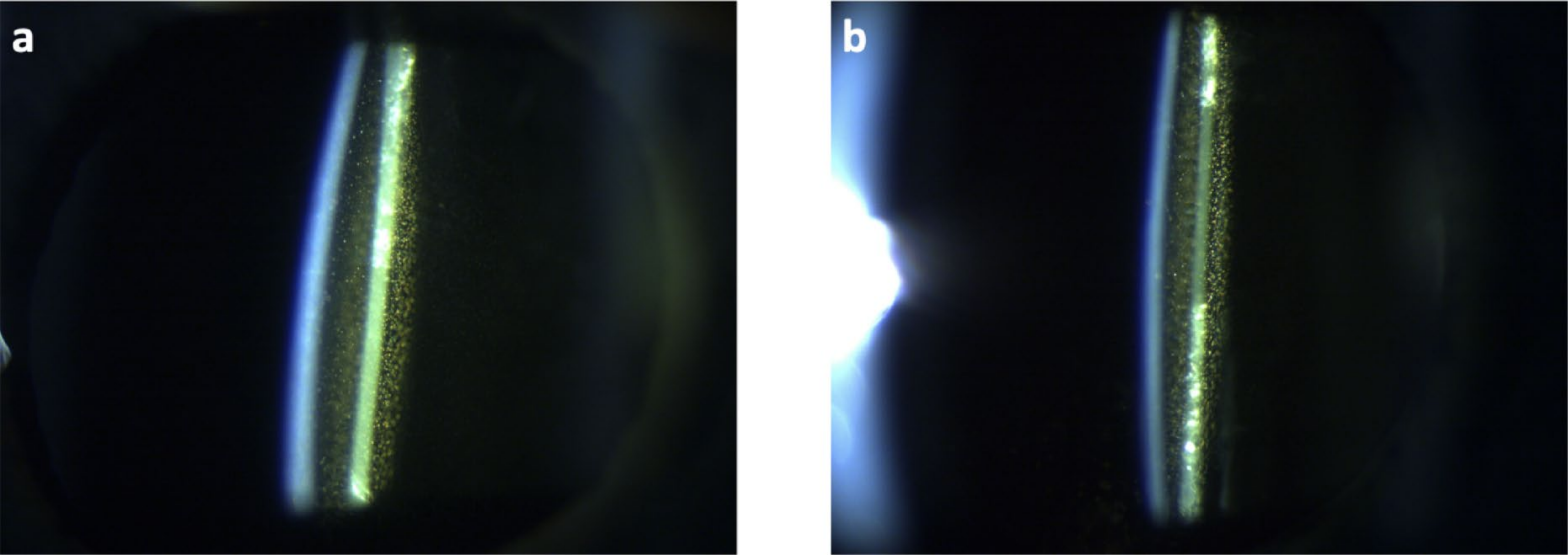
Pre-explantation slitlamp images showing severe glistenings in the SN60 WF IOL in the right (**a**) and left eye (**b**).

We included lenses that were explanted because of intolerable symptoms of glistenings: two AcrySof SN60WF IOLs with a nominal power of 22.5 D and 22.0 D corresponding to the left and right eye of a female patient. The implantations were carried out on March 2010 (OD) and December 2011 (OS). No intra- or postoperative complications were noted. In February 2018, the patient reported photophobia while driving for the past few months. The best corrected visual acuity was 1.2 (OD) and 1.0 (OS), and the best near correction yielded 1.0 in both eyes. The slit-lamp examination using a BQ900 (Haag-Streit AG, Köniz, Switzerland) with 40x magnification, a slit-width of 1 mm angled at 45° with a Haag-Streit imaging module 910 revealed severe glistenings in both eyes (Fig. 2).

The C-Quant measurements indicated increased light scattering, with a logs score of 1.63 in the right and 1.65 in the left eye. After considering the features of the patient’s complaints and characteristics of straylight elevation, it was decided to replace the IOLs with a PodEye G free (PhysIol, Liège, Belgium) IOL for the right eye and Synthesis (Cutting Edge, Montpellier, France) IOL for the left eye. The first explantation was performed on the right eye on January 2020, which was followed by the same procedure in the left eye on June 2021. Implantation and explantation of IOLs were performed at an external center as part of routine procedures. The explants were immediately sent to the David J. Apple International Laboratory for Ocular Pathology (Heidelberg, Germany) for objective evaluation.

### Statistical methods

Statistical analysis was performed with Excel software (Microsoft Corp, Redmond, Washington, USA) and MedCalc statistical software version 23.0.2 (MedCalc Software, Ostend, Belgium). Mean values of the morphological glistenings analysis and the straylight evaluation were calculated for each IOL model. The average values were expressed as the mean ± standard deviation (SD). Then, correlation parameters were evaluated between results of the three methods by using linear regression analysis. Furthermore, Kruskal-Wallis test for multiple groups was carried out. Post-hoc analysis was also added if p-value was < 0.05. Since there were only two IOLs in the explanted IOL group, these IOLs were excluded from the Kruskal-Wallis test for multiple groups.

## Results

The results of the 27 IOLs analyzed with the three different methods are summarized in Table 1. Table 2 shows the mean size of glistenings for each IOL model, which was evaluated with the light microscopy method.

**Table 1:** Summary of the glistening density and straylight parameter of the different analysis methods.

IOL model	Glistening density (1/mm^2^) in light microscopy	Glistening density in SS-OCT (1/mm^2^)	Straylight parameter (deg^2^/sr)
Clareon CNA0T0	1.83 ± 2.66	0.30 ± 0.62	0.98 ± 0.43
Vivinex XY1	3.33 ± 2.20	0.26 ± 0.26	0.78 ± 0.11
MA60AC	249.50 ± 53.30	41.06 ± 7.81	4.48 ± 0.92
SN60WF	208.16 ± 136.11	25.76 ± 17.27	1.63 ± 0.64
PY60AD	2865.16 ± 650.36	212.84 ± 35.49	18.90 ± 5.70
Explanted IOLs (SN60WF)	1369.58 ± 87.79	184.55 ± 39.81	10.65 ± 1.83

**Table 2:** Mean glistening size of each IOL model measured with light microscopy.

IOL Model	Mean Glistening Size ± standard deviation (µm^2^)
Clareon CNA0T0	19.96 ± 0.93
Vivinex XY1	99.11 ± 39.88
MA60AC	103.70 ± 32.15
SN60 WF	63.18 ± 20.35
PY60AD	33.61 ± 4.09
Explanted IOLs (SN60WF)	36.50 ± 0.95

In the first morphological evaluation method using light microscopy, the PY60 AD IOLs had the highest glistening density, ranging from 2052 to 3818 MV/mm^2^, whereas the mean size of glistenings was the smallest in this group. The MA60AC IOLs had the second highest glistening density with the largest mean glistenings size measured in this study. The mean glistening density of the SN60WF IOLs were lower than the MA60AC model, though the distribution of glistening density was less consistent across the tested SN60 WF IOLs, ranging from 60 to 360 MV/mm^2^. In evaluation of the IOL models CNA0T0 and XY1, there were virtually no glistenings detected.

The trend in mean glistening density between the tested IOL models was similar in the second morphological evaluation method with SS-OCT. The PY60AD showed the highest glistening density, followed by the MA60AC and the SN6OWF. The CNA0T0 and XY1 had virtually no glistenings.

The straylight measurements demonstrated that the PY60AD induced the highest amount of straylight, followed by the MA60AC and the SN60WF in a similar trend to the morphological evaluation methods. The CNA0T0 and XY1 had the lowest mean straylight value in this study.

**Fig. 3:**
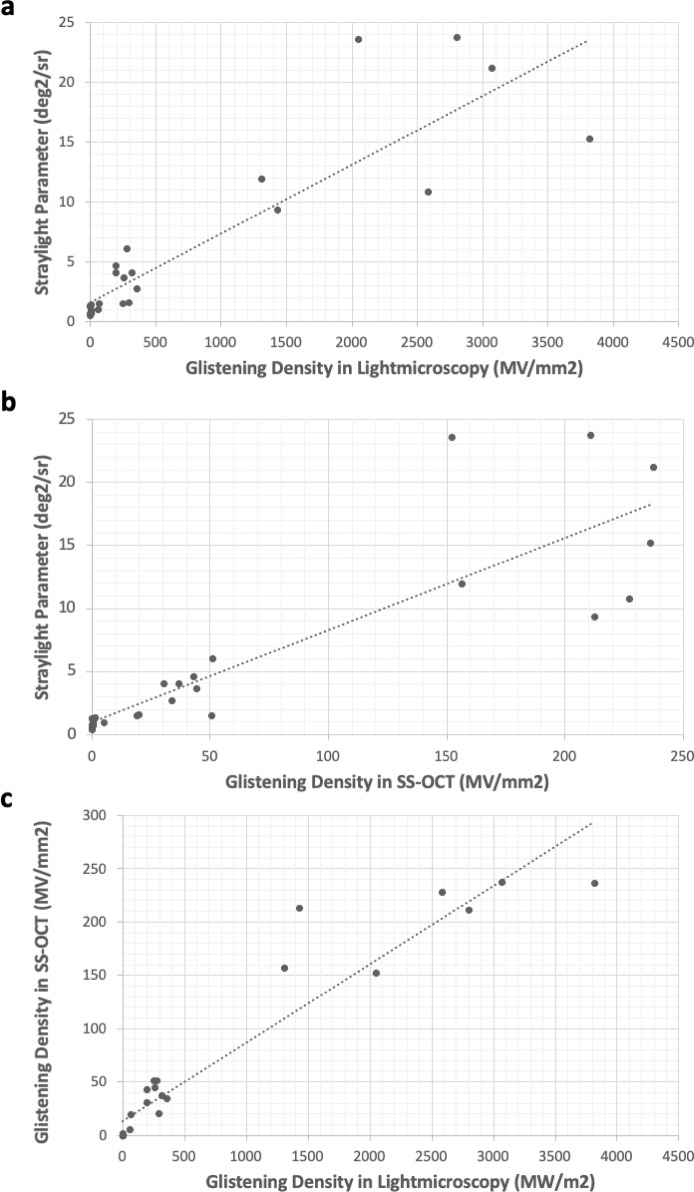
Regression Analysis Between Glistening Density in Light Microscopy and Straylight Parameter (**a**). Regression Analysis Between Glistening Density in SS-OCT and Straylight Parameter (**b**). Regression Analysis Between Glistening Density in Light Microscopy and SS-OCT (**c**).

**Fig. 4:**
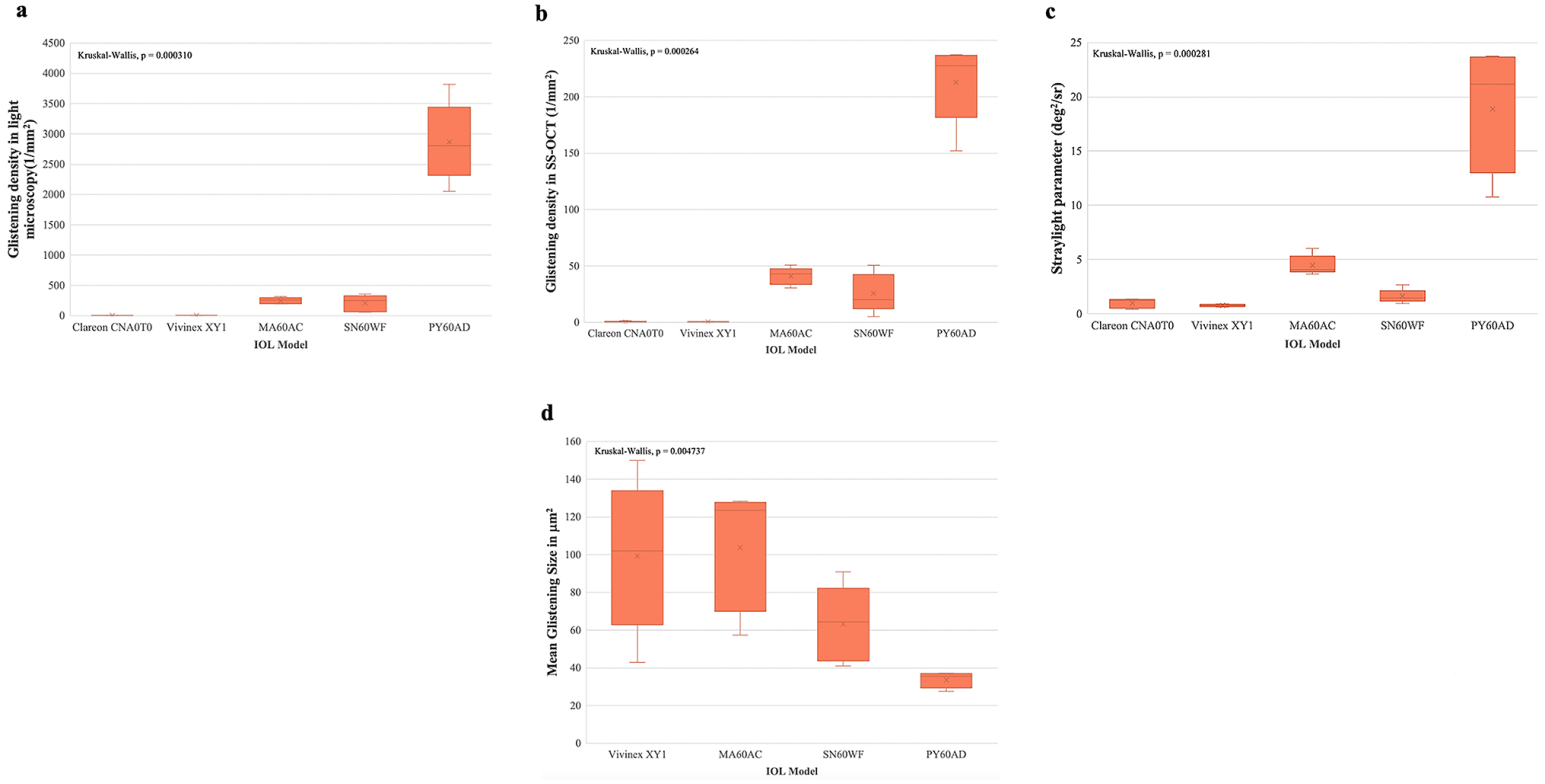
Boxplots depicting the glistening density of IOL models in the light microscopy, SS-OCT, straylight methods including Kruskal-Wallis test results (**a**, **b**, **c**). Boxplot depicting the mean glistening size of IOL models in the light microscopy method (**d**).

The linear regression analysis showed that there was a moderate correlation between the light microscopy and the straylight method with a coefficient of determination (R^2^) of 0.80 (Fig. 3a). The analysis also showed a moderate correlation (R^2^ = 0.77) between the SS-OCT and the straylight method (Fig. 3b). A strong and proportional relationship (R^2^ = 0.91) was found between the light microscopy and the SS-OCT method (Fig. 3c). Furthermore, Kruskal-Wallis test results were given in Fig. 4. Since the p-value was < 0.05 in three analysis methods, post-hoc analysis was carried out. In the post-hoc analysis for light microscopy and SS-OCT methods, the results of the Clareon CNA0T0 and Vivinex XY1 were significantly different from the MA60AC, PY60AD and SN60WF. On the other hand, the results of the PY60AD were significantly different from the Clareon CNA0T0, Vivinex XY1, MA60AC and SN60WF. In the post-hoc analysis for straylight method, the results of all IOL models were significantly different from each other, except for the Clareon CNA0T0 and Vivinex XY1. In the post-hoc analysis for mean glistening size of each IOL model measured with light microscopy, the PY60 AD was significantly different from the Vivinex XY1, SN60WF and MA60AC. The MA60AC was also different from the SN60 WF. The Clareon CNA0T0 group was excluded from the post-hoc analysis for mean glistening size since there were only two IOLs with detected glistenings in this group (Fig. 4).

## Discussion

In this study, five different IOL models with artificially induced glistenings were analyzed with three alternative methods, including light microscopy, SS-OCT and straylight meter. Glistenings were quantified with light microscopy, SS-OCT and straylight meter. The trend was similar in all three examination methods. The PY60AD model showed the highest glistenings, followed by the MA60AC and SN60WF, whereas the CNA0T0 and XY1 showed the lowest values. The proposed approach using SS-OCT demonstrated a proportional relationship with straylight measurements and a very good correlation with the in-vitro reference method light microscopy. Thus, this method has the potential to analyze glistenings in a precise manner and to estimate the straylight induced by glistenings within an IOL.

Compared to other studies which examined the glistening density of different IOL models this study shows similar results. For instance, Łabuz et al. examined different IOL models using light microscopy and straylight measurements. In the examination with light microscopy the glistening density was 3532 ± 340 MV/mm^2^ for the PY60AD, 542 ± 480 MV/mm^2^ for the MA60 AC and 61 ± 33 MV/mm^2^for the SN60WF, whereas the mean glistening size was 5.2 ± 0.4 μm for the PY60AD, 10.2 ± 1.4 μm for the MA60 AC and 8.0 ± 0.6 μm for the SN60WF^7^ Furthermore, the mean straylight parameter measured in the study of Łabuz et al. was 19.30 ± 2.07 deg^2^/sr for the PY60AD, 1.15 ± 0.15 deg^2^/sr for the SN60WF and 5.95 ± 3.67 deg^2^/sr for the MA60AC^7^ These results are very close to the mean straylight parameter we measured for the respective IOL models.

Several in-vivo and in-vitro studies investigated the relationship between glistening density and straylight parameter^6,7,15,22^. This study confirms the proportional relationship between straylight and the total number of glistenings albeit with a slightly lower coefficient of determination (R^2^ compared to the previous studies by Łabuz et al.^6,7^. The coefficient of determination (R^2^) was 0.80 between the light microscopy and the straylight method while R^2^ was 0.77 between the SS-OCT method and the straylight method. However, a strong and proportional relationship existed between the light microscopy and the SS-OCT with R^2^ = 0.91. The experimental plan of this study might have caused that slight decrease of the coefficient of determination between morphological analysis methods (light microscopy and SS-OCT) and functional analysis method (straylight measurement), since, in our workflow, measuring straylight was the last examination method. Even though fluctuation in glistening density was tried to be prevented by using an incubator to minimize temperature changes between stages of examination methods, glistening density might well have decreased with the duration of the experiment and that would potentially produce relatively lower values in the straylight method. It should also be noted that the results of light microscopy and SS-OCT differed, even though the unit of glistening density evaluated with light microscopy and SS-OCT was the same (MV/mm^2^). Notwithstanding the excellent correlation between these two morphological analysis methods (R^2^= 0.91), glistening density in light microscopy was much higher compared to the results in SS-OCT. The resolution of Anterion might have been an important factor causing this difference in glistening density. According to the manufacturer’s specifications, Anterion has an axial resolution < 10 μm and a lateral resolution < 45 μm^23^. On the other hand, the size of a glistening microvacuole can range from 1 to 20 μm^9,24^. The margin is small between the resolution of the device and glistening size, so that the quantity of smaller glistenings might have been underestimated with the SS-OCT contributing to an overall lower glistening count than the light microscopy. This small margin also posed a challenge in the image processing of IOLs with small glistenings, especially the PY60 AD model. Therefore, the images were segmented with TWS before dichotomizing them with a threshold. This allowed to distinguish two adjacent microvacuoles in a precise manner, thus getting more reliable results. In our study, the reliability and reproducibility of this novel examiner independent method with SS-OCT has been proven by strong correlation found with light microscopy method. So, despite the difference in glistening density with light microscopy method, the approach with SS-OCT has the potential to be used to quantify glistenings.

There are reported cases in which IOLs were explanted due to symptoms caused by glistenings. Van der Mooren et al. reported two cases where pseudophakic patients with confirmed presence of glistenings in IOL optics complained about hazy or blurry vision. Following IOL exchange, the symptoms resolved, suggesting that straylight caused by glistenings within the IOL optic might be clinically significant^25^. In this regard, two AcrySof IOLs (SN60WF) in the left and right eye of a patient were included in this study, which were explanted due to glare symptoms caused by glistenings. After explantation, patient’s straylight parameter improved 20.7 deg^2^/sr OD and 16.4 deg^2^/sr OS. So, a more substantial improvement was observed clinically compared to the in-vitro testing of the explanted IOLs which showed an average straylight parameter of 10.65 ± 1.83 deg^2^/sr. The result could potentially indicate that the number of glistenings is more severe when measured in-vivo and could be underestimated in-vitro due to their density fluctuation associated with the temperature change, underscoring the importance of establishing an objective in-vivo method to quantify glistenings. This case also confirms that many patients with earlier-generation hydrophobic acrylic lenses, such as the SN60WF, are still affected by a high number of glistenings. The explanted SN60WF IOLs showed higher values in all the three methods used in our study, when their results are compared to the five SN60WF IOLs with in-vitro induced glistenings. This observation agrees with the findings of Thomes and Callaghan who found a significant reduction in glistening density in the more recent AcrySof model compared to the older generation^3^. Other factors might have caused this difference in glistening density as well. For instance, Miyata et al. observed that the number of glistenings reached its peak after a few months of formation before it became stable^9^. Likewise, Tognetto et al. reported a continuous increase of glistenings in AcrySof material up to 180 days after implantation^2^. Consequently, the five SN60WF with in-vitro induced glistenings could have had lower values than the two explanted SN60WF, since they were evaluated right after the accelerated aging protocol was implemented, whereas the explanted IOLs have had glistenings for a long period of time which was approximately ten years in this case.

So far, glistenings have been analyzed with various in-vivo and in-vitro methods, such as slitlamp examination, Scheimpflug tomography, light microscopy and straylight measurements^9–16,26^. All these methods are proven to have some shortcomings and there is a need for an objective, examiner-independent, in-vivo method. The method introduced in this study is based on the examination of IOL with a SS-OCT device. In fact, glistenings have been examined with OCT-based methods in a few former studies^27,28,17,18^. These studies demonstrated the presence and location of the microvacuoles as a proof of concept^27^. Furthermore, an approach has just recently been suggested by Fernandez-Vigo et al. to quantify glistenings using SS-OCT. In this clinical study, SS-OCT scans of pseudophakic patients were evaluated by a DL-based quantification algorithm^18^. Although the study claims an approach for objective classification of glistenings in IOL, it has a limitation, since the proposed evaluation method was not compared with any of the methods which have been established so far. Hence, there was not a reference method confirming that the quantified hyper-reflective foci (HRF) using SS-OCT were indeed glistenings. However, the proportional relationship between the SS-OCT and the established methods used in this study confirms that HRF quantified by the SS-OCT method could be glistenings. In our study, the maximum available 65 B-Scans along the IOL were taken with the volume scan mode of the SS-OCT device Anterion in the form of an image stack. In this manner, the volume density and distribution of microvacuoles within IOL can be observed clinically as well.

In conclusion, the method proposed in our study has the portential to be used to evaluate the severity of glistenings in patients implanted with hydrophobic acrylic IOLs. The strong correlation observed in glare assessment underscores its significant benefits in accurately estimating the contribution of IOLs to glare, unaffected by any interference caused by straylight originating from other eye parts. The SS-OCT approach can provide an objective measure independent of examiner skills and experience, which with the introduction of a built-in app for scatterers evaluation, may facilitate the implementation of this approach in clinical practice in the future.

## Electronic supplementary material

Below is the link to the electronic supplementary material.

Supplementary Material 1

Supplementary Material 2

## Data Availability

Data is provided within the manuscript.
