# Supplementary material for: A new method for analyzing glistenings in hydrophobic acrylic intraocular lenses
Source: Sci Rep. 2025 May 21;15:17661. doi: 10.1038/s41598-025-00137-9 (PMC12095468; doi:10.1038/s41598-025-00137-9)
Supplement: Supplementary file 2 — Supplementary Material 2 [file 41598_2025_137_MOESM2_ESM.pptx]

## Slide 1
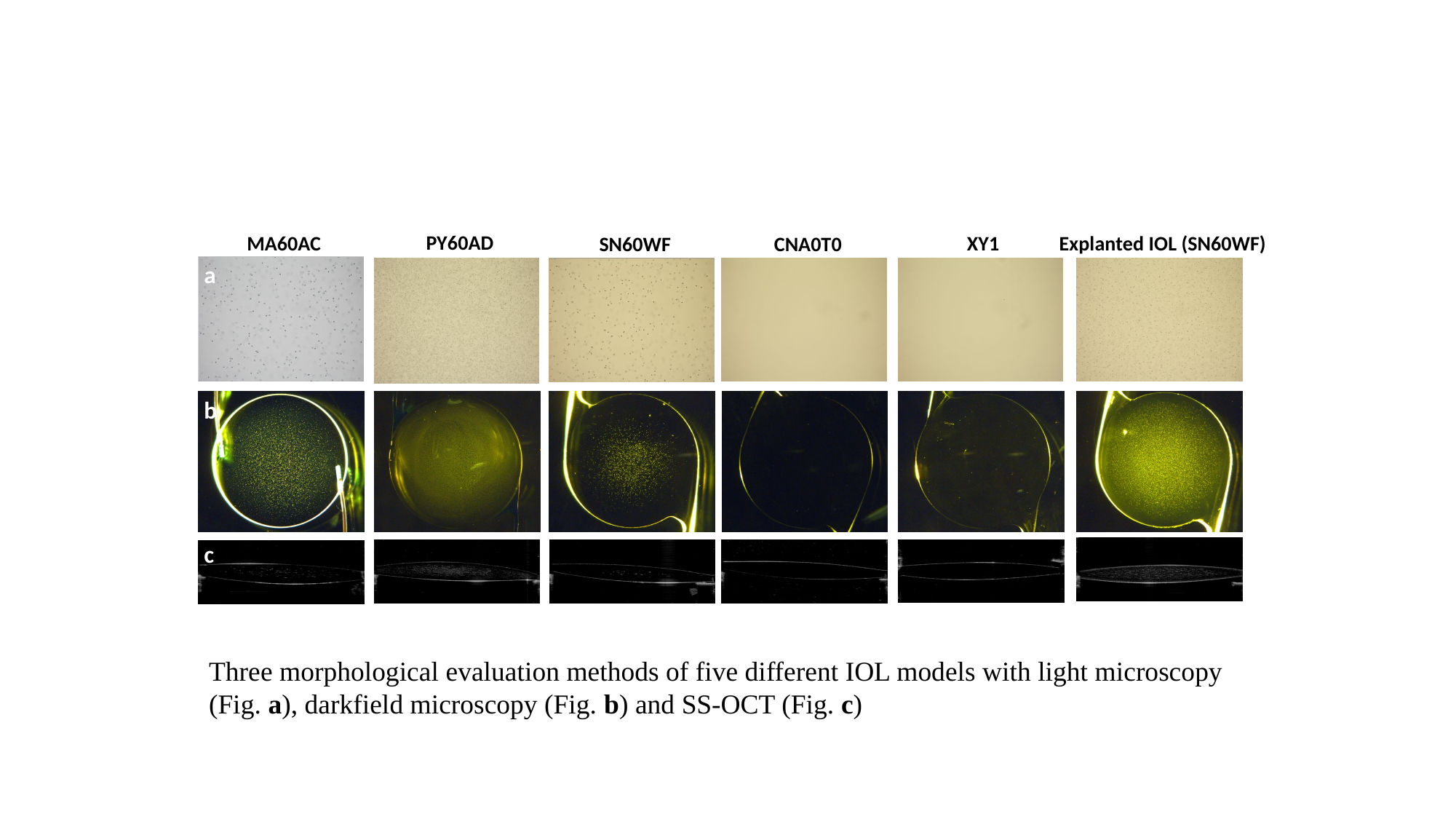

PY60AD
MA60AC
Explanted IOL (SN60WF)
XY1
SN60WF
CNA0T0
a
b
c
Three morphological evaluation methods of five different IOL models with light microscopy (Fig. a), darkfield microscopy (Fig. b) and SS-OCT (Fig. c)
